# Exploring Ayurvedic Knowledge on Food and Health for Providing Innovative Solutions to Contemporary Healthcare

**DOI:** 10.3389/fpubh.2016.00057

**Published:** 2016-03-31

**Authors:** Unnikrishnan Payyappallimana, Padma Venkatasubramanian

**Affiliations:** ^1^Education for Sustainable Development, Institute for the Advanced Study of Sustainability, United Nations University, Tokyo, Japan; ^2^International Institute of Global Health, United Nations University, Kuala Lumpur, Malaysia; ^3^The Institute of Transdisciplinary Health Sciences and Technology, School of Life Sciences, Bangalore, India

**Keywords:** Ayurveda, food and health, ayurbiology, system biology, trans-disciplinary research

## Abstract

Ayurveda, a traditional system of medicine that originated over three millennia ago in the South Asian region, offers extensive insights about food and health based on certain unique conceptual as well as theoretical positions. Health is defined as a state of equilibrium with one’s self (*svasthya*) but which is inextricably linked to the environment. Ayurvedic principles, such as the *tridosa* (three humors) theory, provide the relationship between the microcosm and the macrocosm that can be applied in day-to-day practice. Classical Ayurveda texts cover an array of themes on food ranging from diversity of natural sources, their properties in relation to seasons and places and to their specific function both in physiological and pathological states. The epistemic perspective on health and nutrition in Ayurveda is very different from that of biomedicine and modern nutrition. However, contemporary knowledge is reinventing and advancing several of these concepts in an era of systems biology, personalized medicine, and the broader context of a more holistic transition in sciences in general. Trans-disciplinary research could be important not only for pushing the boundaries of food and health sciences but also for providing practical solutions for contemporary health conditions. This article briefly reviews the parallels in Ayurveda and biomedicine and draws attention to the need for a deeper engagement with traditional knowledge systems, such as Ayurveda. It points out that *recreation* of the methodologies that enabled the holistic view point about health in Ayurveda may unravel some of the complex connections with Nature.

## Introduction

Ayurveda is one of the oldest healthcare systems that evolved in the Indian Subcontinent. From the large number of literature spanning over three millennia on diverse aspects of managing health and wellbeing, both in Sanskrit and regional languages of the subcontinent, it can be deduced that it has had a dynamic and unbroken knowledge tradition (1). Contemporary Ayurveda has been formalized and institutionalized on aspects such as education, clinical approaches, pharmacopeia, and product manufacturing starting from late nineteenth century. In the post independence period in India, it has been recognized and legitimized as one of the formal healthcare systems of the country (2).

**Figure 1:**
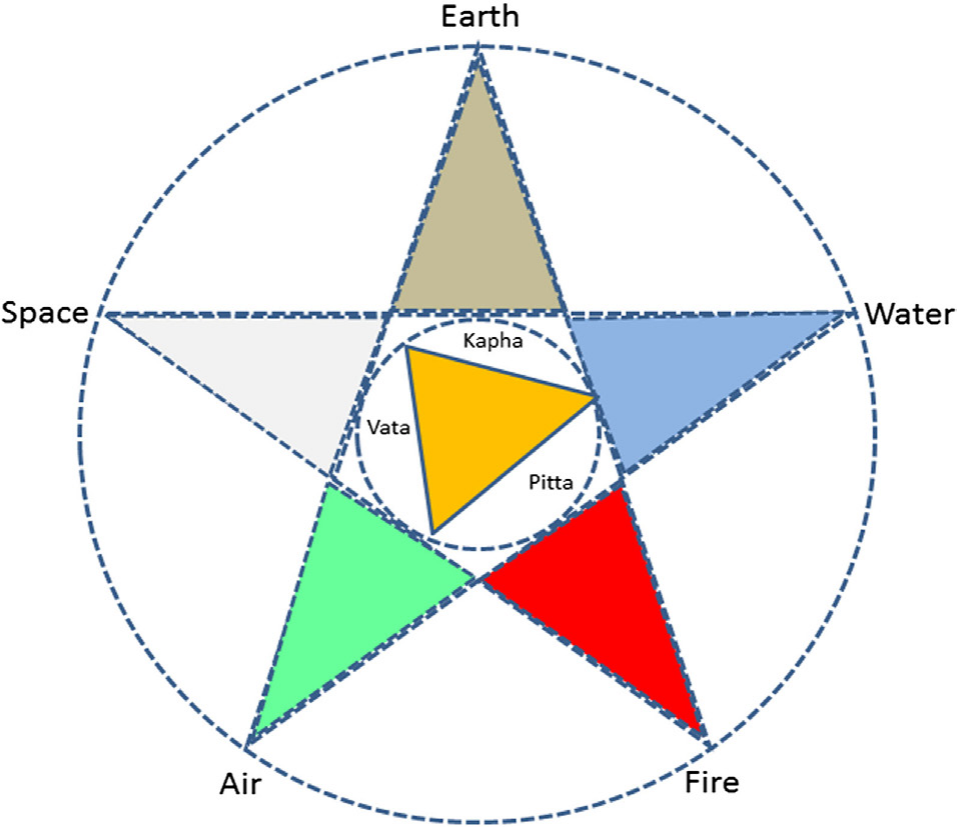
**Panchamahabhutha and tridosa relationship**.

The term Ayurveda comprises two words – *ayu* (life) and *veda* (knowledge), thus, deals with various aspects related to health and wellbeing in their diverse aspects, such as happy life, sustainable happiness, and longevity (3). According to Ayurveda, there are three fundamental states of a being such as the physical (including physiological), mental, and the spiritual. Health is a balance of all these three states and their relationship with the outside world (4). This relationship between the microcosm and the macrocosm is yet another fundamental tenet of Ayurveda. The “being” constantly interacts with the outside world through its senses (senses of knowledge and senses of action) and the cognitive functions. At the same time, the outside world is constantly influencing the being. Both the outside world and the being are understood on the ontological basis of the *pancamahabhuta* or the five element theory. The categorization in terms of the five elements, including earth, water, fire, air, and space corresponds to each of the five senses, *viz*. smell, taste, vision, touch, and sound, respectively (3). This is a fundamental precept of all knowledge traditions in the subcontinent. But in Ayurveda for ease of understanding of physiological and pathological aspects, the five elements are further grouped into three called the *tridosa*–*vata* (a combination of space and air), *pitta* (fire), and *kapha* (water and earth) (Figure 1). Body and mental types, metabolic processes, biological rhythm, seasonal variations, various other physiological and pathological processes, etc. are understood in terms of innumerable permutations and combinations of these elements and the *tridosa* in the body. According to Ayurveda, this forms the basis of understanding of materials (*Dravya guna sastra*), such as food or medicine, therapeutic approaches, and dietary or lifestyle changes, to stay healthy (5). Food classifications based on their organoleptic properties and their impact on psychological constitution of an individual is yet another interesting precept of Ayurveda (6).

While the Ayurvedic concepts and theories may appear quite generic and simplistic in its description, the sophistication is evident from the universality and contemporariness of the applications at the levels of diagnosis and line of treatment. Ayurvedic perspective of Nature and the universe and its interplay with the “being” provides opportunities that can be tapped for applications in modern health sciences and nutrition.

Concerted efforts to scientifically interpret and utilize Ayurvedic knowledge through the eyes of modern science and medicine are ongoing. The first section of the article highlights some of the basic theoretical perspectives of Ayurveda and understanding of health, food, and nutrition, while the second provides a brief overview of the trans-disciplinary research efforts to interpret and use Ayurvedic knowledge. The article introduces some of the Ayurveda concepts and principles that are pertinent to health, food, and nutrition and presents the trends in Ayurveda research.

## Health and Food

*Svasthya*, “to be established in one’s self or own natural state” is optimal health according to Ayurveda. In order to achieve this, one has to have a balance of structural and physiological factors, metabolic and excretory processes, body tissues, senses, mind, and attain a state of self awareness and contented self (6). Ten factors (*dasa vidha pariksa*) are used to determine the state of health of an individual per Ayurveda namely, body tissues (*dusya*), residing location (*desa*), physical strength (*bala*), seasons/time (*kala*), digestive and metabolic processes (*agni* or *anala*), genetic and phenetic constitution (*prakriti*), age (*vaya*), mental strength or temperament (*sattva*), habituation (*satmya)*, and food (*ahara*) (7). It is interesting to note that *sattva* is said to influence health. While these are mainly used for diagnostic purposes, these can also be used to measure the wellbeing of an individual.

### Ahara, a Pillar of Life

*Ahara* is one of the three pillars of life according to Ayurveda; the other two being sleep and regulated sexual life. The classical texts of Ayurveda of 300 BC–700 AD dedicate elaborate sections on foods (6). Unique aspects include detailed descriptions of food and beverage, food classification based on their taste, therapeutic qualities, etc., food safety and measures for the same, different incompatibilities of food based on their tastes, processing, dose, time, place, etc., prescriptions of consumption, food qualities and intake based on the digestive ability of an individual, and the nature of food that is being consumed (3). Primary classification of food is based on its appropriateness to body and mental constitution based on the five elements and the *tridosa* theories. Five elements combine and dissociate in the natural transformation of any material, living or non-living (8).

#### Taste (Rasa) as an Indicator of Health Effects

One of the ways of food classification in Ayurveda is based on *rasa*. There are six major tastes according to Ayurveda, *viz*., sweet, sour, salty, pungent, bitter, and astringent. There are primary and secondary qualities (*guna*) that increase the properties of a material. This is further augmented by potency (*virya*), post digestive effect (*vipaka*), and therapeutic action (*karma*). According to Ayurveda, the classification based on *rasa, guna, virya, vipaka*, and *karma* is not only applicable to foods but also for all materials, including medicines, and is dealt with under *Dravya guna sastra* (the science of materials’ properties), the Ayurvedic pharmacology (8).

#### Food Can Alter Moods

Another interesting taxonomy of foods is based on their effect on psychological dispositions of individuals. According to Ayurveda, there is a subtle link between disease manifestation and the six psychological expressions, such as lust, anger, greed, desire, attachment, and ego. These psychological states are closely linked to foods. This connection is further discussed in terms of three states of being including *sattva, rajas*, and *tamas*. *Sattva* is the contented state, *rajas* an excited state whereas *tamas* relates to a lethargic disposition, i.e., foods can induce these states of mind (9, 10).

#### Proper Metabolism is Key to Good Health

The energy that drives metabolic processes in the body is called *agni*, which also has an important effect on health. There are three stages in the digestive cycle starting from the gross form in the gastrointestinal tract followed by tissue-specific metabolism and elemental level metabolism. In this sequence of events, *vipaka* has a specific impact on the body. Generally, the predominant taste of the food material remains in the post digestive effect; but for materials with multiple tastes, the taste changes post metabolism: an important indicator of its impact on the system. For example, Indian gooseberry (*amla*) is predominantly sour in taste but post-digestive effect is sweet. Even though sour taste can increase *pitta* in the body, gooseberry nullifies *pitta* due to the sweet post digestive function (11). Further according to Ayurveda the action caused by a material (*dravya*) varies depending on the substratum (*dhatu*) and the contextual factors like place, time, and so on (6).

#### Food and Physiological Processes and Actions

In Ayurveda, food has been classified based on morphological features and their corresponding physiological actions. For example, grains, pulses, processed foods, meat and products, leafy vegetables, fruits, salts, supplements, various forms of water, milk and milk products, oils, and alcoholic drinks have been elaborated based on their effect on the body. This is further elaborated in terms of place of origin and seasonal variation (12). Food processing is a topic that is dealt with in detail. Properties of raw, dried, smoked, grilled, pickled, steamed foods, various additives and adjuvants find mention based on the *pancamahabhuta* theory. The pharmacological properties of a substance get altered depending on the processing. For example, puffed rice is light on the system as compared to flaked or cooked rice that is heavy to digest (13). Curd, which is unwholesome in most situations, becomes a healthy drink when churned and the butter is removed. This sweet tasting buttermilk kept in an earthen vessel for 2 days develops astringent taste and becomes a wholesome food for the gastrointestinal system especially in conditions such as hyperacidity, irritable bowel syndrome, fissures, hemorrhoids, and certain types of diarrhea and dysentery (14).

#### Prakriti – A Guide to Personalize Diets

An individual’s *prakriti* is another important determinant of the effect of food on the system. *Prakriti* of an individual is characterized by a set of physical, physiological, and psychological attributes. For example, based on taste preference, individuals can be grouped as *vata* (having affinity for sweet, sour, and salty tastes); *pitta* (with liking for sweet, bitter, and astringent taste), and *kapha* (for pungent, bitter, and astringent tastes). Whereas these tastes mitigate any negative effects of the inherited constitution, usage of tastes in the reverse order can cause imbalance in the body. For example, if a *vata* constitution person continuously consumes pungent, bitter, and astringent tasting materials, it could lead to rapid aging and degeneration of the body (3, 15).

#### Incompatible Foods (Viruddha Ahara) and Processes

Yet another distinctive feature of Ayurveda is its understanding of incompatibilities of food materials and processing. There are 18 forms of incompatibilities according to Ayurveda (3). Incompatibilities are explained based on the potency of materials, processing, quantity/dose, process of intake, time/season; combining materials, such as sour fruits and milk or honey and ghee (clarified butter) in equal quantities; milk along with horse gram, jack fruit, or fish; or even heating honey. Whereas we may not have a contemporary scientific explanation for these, this can be explained in Ayurvedic terms as incompatibility in the nature of the materials.

#### Wholesome and Unwholesome Food (Pathya and Apathya)

Several examples are provided in Ayurvedic texts in terms of wholesome supplements, *pathya* and *apathya*. These are particularly indicated in disease management. For example, pomegranate, amla (Indian gooseberry), buttermilk, etc. are mentioned as good *pathya ahara* in the management of iron-deficiency anemia (16). Processing of a material can change the potency, safety, and pharmacological effect of the material. Curd is considered unwholesome in most *dosa* imbalanced conditions. There are specific instructions to consume yogurt; that it should not be taken at night, or in seasons such as spring, summer, and fall. It should be taken with sugar candy or green gram soup or honey. There are also disease-specific or medicine-specific instructions that should be followed for consumption of food. As an example, a patient suffering from cough is advised to consume vegetables, such as coccinea; spices such as garlic and cardamom, long pepper, ginger, and condiments prepared with puffed paddy (16). It is indicated that certain tastes have direct correlation with disease manifestation, hence, they are avoided during treatment of those conditions. There are also detailed descriptions of convalescence foods (17).

From the above section, it is clear that Ayurveda has its own universally applicable principles, methods, and practices that are very different from biomedicine and modern nutrition concepts (Table 1). Several efforts are ongoing to understand and interpret Ayurveda on the basis of contemporary science. A recurring criticism has been that natural science research in Ayurveda has been not adequately effective and has been limited to ethnobotany, biochemistry, and pharmacology. Most research initiatives till last decade were restricted to looking for compounds or molecules from Ayurveda drugs that could be absorbed to the modern pharmacopeia. These have not resulted in any substantial contributions in terms of new diagnostic techniques, formulations, or treatment approaches in Ayurveda except for some minor outcomes of validating certain Ayurvedic practices. This illustrates a distinct need to focus on fundamental research based on Ayurvedic principles for identifying new pathways and for more holistic perspectives in preventive as well as curative healthcare. There is much to be researched into the unique Ayurvedic concepts in nutrition, including *pathya, viruddha ahara*, *kala* and *desa vicara*, *agni*, *ama*, etc. (6, 18).

**Table 1:** **Epistemological comparison of modern biomedicine and Ayurveda**.

	Aspects	Modern biomedicine	Ayurveda
1	Approach and disease classification system	Largely focuses on structural/material aspects	Mainly focuses on functional aspects
2	Location	Organ specific or localized	Systemic
3	Causality	Single Causality	Multiple causality
4	Reasoning method	Linear	Non-linear, circular reasoning
5	Causative reason	Largely organism centered/external	Primarily immunity centered
6	Nature of knowledge	Objectivity centered	Subjectivity centered
7	Nature of assessment	Predominantly quantitative	Predominantly qualitative
8	Context of validation	Outside individual’s context, laboratory	Within the context
9	Diagnostic approach	Universalization of standards	Individualization
10	Domains	Physical and mental; disease centered	Physical, mental, and spiritual; Illness centered
11	Treatment focus	Curative focus, importance given to drugs, surgery	Preventive and promotive focus, importance given to drugs, food, and lifestyle
12	Treatment strategy	Targeted medicine	Compound formulations (*yoga*) and line of treatment concept
13	Line of treatment	Treating a specific manifestation at given time	Stage-wise management of the illness
14	Outcome	Effect is important	Effect should not lead to after effect, quality of life
15	Knowledge/practice focus	Standard protocols and institution driven	Physician driven

## Bridging Trans-Disciplinary Research

There have been several ethnobotanical explorations into Indian health knowledge systems by many western and Indian enthusiasts. *Hortus malabaricus*, a seventeenth-century-AD Dutch publication is an example that contains 12 volumes of information about the plants and the local uses from Malabar (better known as Kerala). The “Wealth of India,” an encyclopedic series, was published through meticulous documentation and research by scientists in national research institutions on economically important medicinal plants in the post independent India. Sporadic research efforts continue on the botanical, chemical, molecular, and pharmacological aspects of Indian medicinal plants and traditional medicine. However, in the last decade or so there has been a renaissance of sorts of Ayurveda with renewed interest in bridging Ayurveda and science, to understand and interpret Ayurvedic concepts, i.e., this interest goes beyond validating the use of medicinal materials *per se* to specifically unravel the concepts in Ayurveda. “A science initiative in Ayurveda” authored by M.S. Valiathan has sown the seeds by bringing in for the first time, a scheme called “Ayurvedic Biology” under the premier, mainstream, national funding agency, the Department of Science & Technology. (http://dst.gov.in/whats_new/whats_new12/AYURVEDIC%20BIOLOGY.pdf). Several concepts, including *prakriti, dosa, rasayana*, and *panchakarma*, are being researched by leading scientific institutions in the country as co-ordinated projects. Publications on research in Ayurveda can be classified as below.

### Mainstream Ayurveda Research

Drug discovery, quality standardization, pharmacological, and clinical validation of the safety and efficacy of medicines have been the mainstream focus areas of Ayurveda research. Pharmacological benefits of many Ayurvedic plants and formulations are being rigorously studied and validated scientifically to be exploited in the treatment of diseases. Even though this approach was being practiced since the early twentieth century, it has gained new impetus subsequent to the christening of the approach as “Reverse Pharmacology.” Reverse pharmacology is “the science of integrating bedside documented experiential hits into leads by trans-disciplinary exploratory studies and to further develop these leads into drug candidates by state-of-art experimental and clinical research” (19, 20) Some well studied plants using the reverse pharmacology approach have been Turmeric (*Curcuma longa*), Asvagandha (*Withania somnifera*), Brahmi (*Bacopa monnieri*), Sarpagandha (*Rauwolfia serpentina*), Neem (*Azadirachta indica*), Guggulu (*Commiphora mukul*), Guduchi (*Tinospora cordifolia*), Indian gooseberry (*Phyllanthus emblica*), Kizhanelli (*Phyllanthus amarus*), Pomegranate (*Punica granatum*), Vidanga (*Embelia ribes*), and Bakuchi (*Psoralea corylifolia*). Compounds isolated from a few of the Ayurvedic plants have gone on to becoming drugs or nutraceuticals, such as reserpine, embelin, azadirachtin, and curcumin (21). In India, New Millennium Indian Technology Leadership (NMITLI), the partnership program of premier national institutions and industry, led to the scientific exploration of Ayurvedic formulations to develop drugs for arthritis, diabetes, and psoriasis (20).

Official pharmacopeias, including the Indian Pharmacopeia and Ayurvedic Pharmacopeia of India bring out monographs that provide botanical and chemical characterization of herbs and formulations as per routine modern scientific methods. Finer aspects of traditional wisdom regarding the quality, including time and place of collection rarely get reflected in these monographs. However, traditional knowledge guided quality standardization of herbal medicines was proposed as a better strategy showing a few preliminary experimental findings (22, 23), calling the approach as Reverse Pharmacognosy (24). Despite Ayurvedic treatments being more than a drug-based therapy, including diet, massage, yoga, etc., until recently, Ayurvedic medicines were put through the same clinical trial protocols as modern drug trials. However, recent thoughts regarding whole system trials call for a different approach that can do justice to holistic Ayurvedic treatments (25, 26).

## Research into Ayurvedic Concepts

It was being increasingly recognized by Ayurveda scholars and Ayurveda-Science enthusiasts that not much was being done to unravel the concepts in Ayurveda (27, 28). Certain efforts are opening up new dialogs with Ayurveda, bioscience, and biomedicine, some of which are listed below. The example below is just to indicate the ongoing trans-disciplinary research trends and is not intended as a comprehensive review of published literature.

### Ontology and Theories

Ontology is a systematic account of existence. In Ayurveda, the method of “knowing” through experimentation and direct evidence can be compared to “*Pratyaksa pramana*,” which is only one of the four ways of “knowing.” *Aptopadesa* (knowledge gained from certain individuals who have foregone material needs), *Anumana* (observation), and *Yukti* (logical conclusion from observed phenomena) are the three other ways (3). Galilean and Cartesian philosophies adopt reductionist methods and experimental verification, which are the premise of biomedicine (29). The main challenge in comparing the ontologies of the two systems pertains to the lack of appropriate vocabulary to share and interpret (30). Also, the lack of exact comparable entities owing to different epistemologies of the two systems may be the other hurdle. The exact biomedical definition of terminologies, such as *Pancamahabhuta*, *tridosa*, i.e., *vata, pitta*, and *kapha*, *dravya guna sastra*, *prakriti*, *agni*, *ama*, and several others, does not exist today, nor can they be likened to a single entity (18).

A glossary of Ayurvedic terms used in the article has been provided in Table 2 to provide a gross level understanding.

**Table 2:** **Glossary of Ayurvedic terms**.

Ayurvedic terms	Meaning
*Agni/anala*	Energy driving transformations in the body, e.g., digestive and metabolic processes
*Agnivyapara*	Regulation of *agni*
*Ahara*	Food
*Ama*	Un-metabolized materials
*Amla*	Sour taste
*Anumana*	knowledge gained through inference
*Apathya*	Unwholesome
*Aptopadesa*	Knowledge gained from certain individuals who have relinquished material needs
*Ayu*	Life
*Bala*	Physical strength
*Dasa vidha pariksa*	Ten factors used to determine the state of health of an individual
*Desa*	Residing location
*Desa vichara*	Theories on the effect of geographical location on life/physiology
*Dhatu*	Substratum/tissue
*Dosa*	Humors or bio-effectors viz. *vata, pitta, and kapha*
*Dravya*	Material
*Dravya guna sastra*	Ayurvedic pharmacology
*Dusya*	Body tissues
*Guna*	Qualities of a material
*Jvara*	Fever
*Kala*	Time/seasons
*Kapha*	One of the three humors; a combination of water and earth
*Karma*	Therapeutic action of a material
*Kasaya*	Astringent taste
*Katu*	Pungent taste
*Lavana*	Salty taste
*Madhura*	Sweet taste
*Medhya rasayana*	Ayurvedic nootropics
*Panchakarma*	Five therapies of Ayurveda that helps in establishing homeostasis in the body. They are emesis, purgation, medicated enemas, oil enema, and nasal medication
*Panchamahabhuta*	Five elements viz. *prithvi* (earth), *jala/ap* (water), *agni* (fire), *vayu* (air), and *akasa* (space)
*Pathya*	Wholesome
*Pitta*	One of the three bio-effectors; mainly made up of fire
*Posana*	Nourishment to tissues
*Prakriti*	Genetic and phenetic constitution
*Pratyaksa*	Knowledge gained through experimentation and direct evidence
*Rajas*	Excited state of mind, which is one of the three mental states
*Rasa*	Taste of a material
*Rasapanchaka*	Five parameters, which determine the therapeutic action of a drug
*Rasayana*	Rejuvenative methods
*Satmya*	Habituation
*Sattva*	Contented state of mind/mental strength or temperament
*Sad rasas*	Six tastes
*Srotosodhana*	Clearing the macro and micro channels
*Svasthya*	A state of equilibrium with one self
*Tamas*	Lethargic disposition of mind, which is one of the three mental states
*Tikta*	Bitter taste
*Tridosa*	Three humors or bio-effectors viz. *vata, pitta*, *and kapha*
*Vata*	One of the three humors; a combination of space and air
*Vaya*	Age
*Veda*	Knowledge
*Vipaka*	Post digestive effect of a material
*Viruddha ahara*	Incompatible foods
*Virya*	Potency of a material
*Yukti*	Logical conclusion from observed phenomena

### Ayurvedic Biology and Systems Biology

Ayurvedic principles are largely holistic and based on an epistemology that is very different from modern biomedicine (Table 1), which is more reductionist in its approach. The ontologies of the two systems are different as mentioned above. The methods and instruments used in biomedicine are designed to understand genes, atoms, molecules, cells, tissues, and organisms. Well-disciplined and *calibrated* sensory organs and mind were used as instruments in Ayurveda to perceive, understand, and theorize fundamental unifying patterns in Nature. From a theoretical point of view, focus on systemic and functional aspects of health and diseases; multi-causality approach; a circular method of cause–effect reasoning; subjective, qualitative, individualized, personalized, and immunity-centered, stage-wise management; and attribution of importance to physicians’ wisdom are a few notable features of Ayurveda and other traditional medical systems (31). The focus in such a management approach is not only physical but also mental and spiritual. This is quite different from the biomedical approach that is largely structural; organ specific and localized; single causality approach based on linear reasoning; organism centered; objective; quantitative; universalized; mostly curative; and drug-based targeted medicine approach. Whereas the Ayurvedic approach is individual physician centered as against the institutional approach of modern medicine (31). New species of plants or diseases or management methods are incorporated into the repository of knowledge as and when they are understood on Ayurveda principles and utilized. Specific focus on side effect along with effect is of high concern in the Ayurvedic approach as evidenced in the design of polyherbal formulation containing a main drug and supportive drugs.

Ayurveda has a top-down, holistic understanding of organisms and their interactions with the environment at a functional level and not so much at the molecular level. The practical application of the *tridosa* theory, for example, is grossly valid across viewing and understanding Nature, balancing health by using natural materials and in diagnosis and line of treatment of diseases (32). Biomedicine, on the other hand, has a bottom-up approach that understands the structural basis and molecular processes of cells, tissues, and organs and a not so much of the functional networks within a system and its interactions with the environment. The current day Systems Biology approach aims to be more holistic but is also derived from a systematic synthesis of data obtained from the reduced view of a system, using extensive computational and mathematical modeling of the inter-linkages.

### Prakriti and Ayurgenomics

Ayurveda has a unique way of classifying humans, which is used in the clinical management of health and disease. Humans are classified into three fundamental types of constitution or *prakriti*, called *vata, pitta*, and *kapha* based on their anatomical, physiological, and psychological characteristics. According to Ayurveda, *prakriti* of a person is determined at the time of conception and does not change until death. Recommendations on diets, lifestyles, and drugs vary depending on the *prakriti* of the individual (3). Since it gets determined at the time of conception, in the past decade, the hypothesis that *prakriti* has a genetic basis was tested by different groups of Indian scientists. A correlation between specific *prakriti* and HLA-DRB1 polymorphism was demonstrated by Bhushan et al., (33). Prasher et al. (34) and Mukherjee and Prasher (35) have used *prakriti*-based classification and have demonstrated the genomic and biochemical correlates with specific *prakriti* types. They have termed this approach of classification of humans as *Ayurgenomics* and propose its potential use in personalized and preventive medicine. Frequency of association of CYP2C19 genotype was demonstrated to vary depending on the *prakriti* (36). Differential expression of a high-altitude adaptation gene, *EGLN1* as a response to hypoxia, was correlated to specific *prakriti* type (37). Rotti et al. (38) found a significant correlation between dominant *prakriti* to place of birth and body mass index (BMI).

### Rasayana

*Rasayana* is a branch of Ayurveda that deals with methods to enhance quality of life and extend lifespan. It mentions plants, animal products, metals, and minerals that can be used as *Rasayana* materials to delay aging and enhance wellbeing. It also describes the non-material-based methods, including meditation, good thoughts, conduct, and lifestyle that can influence well being. *Rasayana*s are useful in preventive, promotive, and curative health. They are said to mainly act in three possible ways (i) *Agnivyapara*: by regulating *agni* that drives transformations in the body, like the metabolic processes, (ii) *Srotosodhana*: by clearing the macro and micro channels thereby increasing tissue perfusion, and (iii) *Posana*: by nourishing tissues (39, 40). Apart from tissue nourishment or perfusion, it is also to create balance of *dosa* that facilitates adequate space, optimal movement and degeneration, regulated metabolism/transformation, and optimal softness and hardness of the physical structure. According to Ayurveda, if the above three are streamlined, the body is maintained in optimal health (1). Even though this makes a lot of common sense at the gross level, it is a challenge to test these concepts in its entirety.

Research into *Rasayana* is at a heightened state today due to the contemporary interest in anti-aging, rejuvenation, and focus on wellness. Age-related diseases, such as Alzheimer’s, Parkinson’s, arthritis, atherosclerosis, and so on, can benefit by probing the concept of *Rasayana*. Contemporary bioassay tools such as *in vitro* and small organisms such as Drosophila, *Caenorhabditis elegans*, yeast are now being used to observe the physical, physiological, and behavioral effect of *Rasayana*s and gain insights into the molecular mechanisms of action (41). Several studies have shown that *Rasayana* plants have anti-oxidant, adaptogenic (42), anti-inflammatory (43–45), anti-cancer (46), and anthelmintic (47) activities. Pomegranate, a *pathya* fruit according to Ayurveda was shown to enhance the lifespan and health span in the fruitfly (*Drosophila melanogaster*) model (48). Pippali (*Piper longum* L.) and amla (*Phyllanthus emblica*) were shown to be effective bioavailability enhancers due to the inherent ability to increase *agni* and thereby digestion and absorption. *In vitro* cell free and cell (Caco-2 and HepG2)-based models (49) were used as bioassay tools. Pharmacokinetics and dynamics of ­piperine – an active constituent of Pippali is also reported (50, 51). Pre-clinical studies on *Medhya Rasayana* (Ayurvedic nootropics) such as Asvagandha, turmeric, and brahmi have demonstrated the ability to clear amyloid-β plaques in neurons that are observed in Alzheimer’s disease (52). Research into *Rasayana* holds the potential to unravel diet and herbs-based preventive methods to curb neurodegeneration and aging, in general.

### Dravya Guna Sastra

*Dravya guna Sastra* literally means “material properties science.” Ayurvedic pharmacology classifies materials based on the sensorial and pharmacodynamic effects on the body. *Rasa*, *guna*, *virya*, and *vipaka* determine the *karma* that a drug or material would have on the body. Not much research has gone into furthering the trans-disciplinary understanding of the five parameters (*Rasapancaka*) that classify a *dravya*. However, there are a few commentaries and hypotheses drawing parallels. In Ayurveda, rasa is said to be the total sensory experience involving the nose, tongue, and throat, based on which six types of *rasa*s have been defined, namely *madhura*, *amla*, *lavana*, *katu*, *tikta*, and *kasaya*. A well-balanced Ayurvedic diet would always have the six tastes (*sad rasas*), which in turn plays a role in homeostasis. Excessive intake of one or the other *rasas* can tilt the equilibrium leading to a health problem. As an example excessive intake of sweet tasting foods can lead to obesity, pungent tasting foods to acidity, etc. (6, 53).

“The strength, complexion, immunity etc. of a living system is under the control of diet which in turn is under the control of six *rasa*s” – Susruta Samhita, Sutra Sthana, Chapter 1, verse 28 (54).

Also, *dosa* imbalances in the body can be countered using materials with specific tastes. In *jvara* (fever), there is an aggravation of *pitta dosa* and is countered by bitter tasting drugs that pacify *pitta* like *Andrographis paniculata* or *Azadirachta indica* (5).

The four main tastes recognized in modern bioscience are sweet, sour, salty, and bitter (55). Conventional bioscience does not recognize pharmacological attributes to tastes. Only in the last decade, there have been studies showing the possibility of a correlation between taste and pharmacology. Beauchamp et al. (56) correlate the anti-inflammatory property to the pungency of oleocanthal (a compound found in olive oil) and ibuprofen (56).

Joshi et al. (57) hypothesize that the possible combinations of *rasa* in Ayurveda probably correlate with specific enzyme active sites in the body. They speculate that the number of distinguishable tastes formed by the permutation-combination of the six tastes is more than enough to distinguish the molecular shapes binding to all enzyme active sites in the body. However, in 1993, Shallenberger rejected the role of taste in any enzymatic reaction because he says that unlike in enzyme reaction there is no change in the substrate during taste perception. Palmer (58) looks at taste as a chemical inducer of signal transduction by binding to G-protein-coupled receptors (GPCRS) and proposes taste signaling as an amenable tool to the methods of pharmacology. The review by Rath et al. (59) on the scientific basis of rasa discusses the importance of structure–function relationship of chemicals but stops short of correlating it with *rasa*s.

### Network Pharmacology and Ayurvedic Network Pharmacology

The next paradigm in drug discovery is touted to be *network pharmacology*. Designer drugs with exclusively selective ligands to avoid side effects may become a thing of the past because a growing body of knowledge in the post-genomic biology era is revealing that drug action cannot be simplified to one drug–one target–one disease theory as understood earlier. It is more complex than a simple lock and key mechanism. There could be several “locks” (drug targets) opened by the same “key” (drugs) and several keys that can open the same lock (60, 61). Traditional medicine-based polyherbal formulations have been studied using -omics technologies and proposed as new strategies in drug discovery instead of single molecule-based drugs to tackle disease complexity (62, 63). In a recent publication, the method and concept of network pharmacology were extended to the analysis of a classical Ayurvedic formulation to understand the rationale and putative synergistic actions of ingredients in a traditional formulation (63).

## Conclusion

Ayurvedic theories and practices on health, food, and nutrition are quite different from those of biomedicine and modern nutrition. Systematic exploration can provide new insights to health and nutritional sciences to provide contemporary solutions in healthcare, for instance, how one can modulate the diet and lifestyle to suit one’s *prakriti*, age, and season. *Rasayana* in particular is an area worth exploring for new ways of rejuvenation and anti-aging. Healthcare costs are a major concern to the government exchequers of both developing and developed countries. Knowledge as to how to manage health at an individual level can help bring down sky rocketing healthcare costs through providing wellness. The Ayurvedic principles and practices can potentially become relevant for designing an integrated health care strategy. Concepts in Ayurveda, such as the *rasa* of a material being an indicator of its action on the body, are new to biomedicine and the modern nutritional sciences and can provide practical ways to create balanced diets.

Two main observations emerged from an analysis of published literature particularly from India on the bridging efforts. There have been more articles hypothesizing correlation of certain Ayurvedic concepts with biomedical entities than actual scientific studies to illustrate the correlation. Second, wherever there have been experimentation and scientific studies to prove the hypothesis, an Ayurvedic concept or an entity has been correlated with an already existing/evolving biomedical or science paradigm rather than introducing a new paradigm from Ayurveda. This approach appears somewhat “forced,” may be because the bridging effort is being attempted by mainstream scientists and/or just because of lack of know-how to study the “holism” of Ayurveda. To state that this approach can only help look at parts of Ayurveda and not the whole is commonplace. However, there is no denying that this is most needed and that this active interest taken by biomedical scientists in Ayurveda will create new knowledge (27).

“I cannot claim that Ayurvedic Biology helps, or will help Ayurveda or Biology. Neither needs help. However, when new science, new techniques are applied to old science, new sprouts of knowledge would appear”

- M.S. Valiathan [eminent cardiologist, medical scientist and a learned scholar of Ayurveda] (64).

In recent years, there have been concerted research efforts to understand Ayurvedic principles, such as *prakriti*, *dosa*, and *agni* using modern scientific tools. These trans-disciplinary bridging efforts have no doubt helped correlate certain reduced aspects of Ayurveda with existing biomedical entities. However, they have not been able to capture the holism of Ayurveda. It is not clear whether the holistic knowledge of Ayurveda was assembled by putting together pieces of data and information gained over a period of time (like is done in Systems Biology approach) or whether there was an altogether different method adopted to perceive holism. Therefore, interpreting or validating the complex holistic principles, such as the five fundamental elements and the three bio-effector concepts (three humors or *tridosas*), of Ayurveda can be quite a challenge using the same methods and tools that look at cells and atoms. This may end up like the five blind men trying to interpret an elephant by feeling different parts of the elephant.

However, the post-modern scientific perspectives and methodologies in systems biology, pharmaco- and nutri-genomics, personalized medicine, chaos theory, ecological human being, etc., are today better equipped to engage in a dialog with Ayurveda than conventional biomedicine. Recreating Ayurvedic methodologies may hold the clue to understand Ayurveda holistically. This is of course not easy and would require a deeper engagement with Ayurveda.

## Author Contributions

UP has contributed the introductory section, sections on the traditional knowledge aspects, and part of the bridging transdisciplinary research section and conclusion. PV has contributed to the introduction section and has written the transdisciplinary research and the concluding sections.

## Conflict of Interest Statement

The authors declare that the research was conducted in the absence of any commercial or financial relationships that could be construed as a potential conflict of interest.
